# *Treponema pallidum* Disrupts VE-Cadherin Intercellular Junctions and Traverses Endothelial Barriers Using a Cholesterol-Dependent Mechanism

**DOI:** 10.3389/fmicb.2021.691731

**Published:** 2021-07-20

**Authors:** Karen V. Lithgow, Emily Tsao, Ethan Schovanek, Alloysius Gomez, Leigh Anne Swayne, Caroline E. Cameron

**Affiliations:** ^1^Department of Biochemistry and Microbiology, University of Victoria, Victoria, BC, Canada; ^2^Division of Medical Sciences, University of Victoria, Victoria, BC, Canada; ^3^Department of Medicine, Division of Allergy and Infectious Diseases, University of Washington, Seattle, WA, United States

**Keywords:** dissemination, *Treponema pallidum*, endothelial transmigration, infection, syphilis

## Abstract

*Treponema pallidum* subspecies *pallidum*, the causative agent of syphilis, traverses the vascular endothelium to gain access to underlying tissue sites. Herein, we investigate the mechanisms associated with *T. pallidum* traversal of endothelial barriers. Immunofluorescence microscopy reveals that a subpopulation of *T. pallidum* localizes to intercellular junctions and that viable *T. pallidum*, as well as a *T. pallidum* vascular adhesin (Tp0751), disrupts the architecture of the main endothelial junctional protein VE-cadherin. Intriguingly, in this study we show that *T. pallidum* traverses endothelial barriers with no disruption in barrier permeability. Furthermore, barrier traversal by *T. pallidum* is reduced by pretreatment of endothelial cells with filipin, an inhibitor that blocks cholesterol-mediated endocytosis. Collectively, these results suggest that *T. pallidum* can use a cholesterol-dependent, lipid raft-mediated endocytosis mechanism to traverse endothelial barriers. Further, treponemal localization to, and disruption of, intercellular junctions suggests that a paracellular route may also be utilized, a dual traversal strategy that has also been observed to occur for leukocytes and other invasive bacteria.

## Introduction

*Treponema pallidum* subspecies *pallidum*, the causative agent of syphilis, is a highly invasive pathogen that crosses the placental, blood-brain, and endothelial barriers. Previous studies have shown that *T. pallidum* enters the bloodstream within hours of infection (Raiziss and Severac, 1937; Cumberland and Turner, 1949) and that *T. pallidum* can penetrate endothelial cell monolayers (Riviere et al., 1989; Thomas et al., 1989). The vascular endothelium is a dynamic cellular barrier that lines the luminal surfaces of blood vessels and separates the circulatory system from surrounding extravascular tissue and organ sites. Endothelial barriers play critical roles in regulating vascular hemostasis as well as innate and adaptive intravascular immune reactions in response to signals of inflammation, damage, or infection. Bacterial pathogens that undergo systemic dissemination to invade secondary infection sites traverse the vascular endothelium to gain access to underlying tissue sites. There are two recognized routes for transendothelial migration: transcellular traversal in which cells and pathogens induce endocytosis to cross the barrier, and paracellular traversal whereby cells and pathogens move in between cells at the sites of cellular junctions to access extravascular areas (Lemichez et al., 2010).

While it is known that *T. pallidum* can invade diverse anatomical sites within the host, the molecular mechanisms that underlie the process of *T. pallidum* transendothelial migration are poorly understood. *In vitro* evaluation of *T. pallidum* interactions with aortic endothelial cells demonstrates that treponemes localize to intercellular junctions and traverse monolayers without disrupting barrier integrity (Thomas et al., 1988). Conversely, scanning electron microscopic imaging of *T. pallidum* interacting with brain microvascular endothelial cells reveals that treponemes can merge with endothelial membranes (Wu et al., 2017). These findings imply that *T. pallidum* may be able to use both a paracellular route and a transcellular route for *T. pallidum* transendothelial migration.

Herein, we explore the mechanisms of *T. pallidum* endothelial barrier traversal. Immunofluorescence microscopy demonstrated that a recombinant version of the only identified *T. pallidum* vascular adhesin Tp0751, as well as live *T. pallidum*, disrupts the architecture of VE-cadherin, the main endothelial junctional protein which plays a key role in regulating vascular permeability. Furthermore, a subpopulation of *T. pallidum* was found to localize to intercellular junctions. Despite the observation of junctional reorganization in endothelial cells, no changes to endothelial barrier permeability were observed in recombinant Tp0751- or *T. pallidum*-treated monolayers in transwell plate assays. Further investigations revealed that viable *T. pallidum* exhibited a low but detectable level of endothelial barrier traversal, in line with prior investigations (2). Finally, *T. pallidum* transendothelial migration was significantly reduced by the addition of an inhibitor that blocks cholesterol-mediated endocytosis, but no change in traversal was observed in the presence of inhibitors of macropinocytosis or dynamin-dependent endocytosis. Collectively, these results suggest that *T. pallidum* uses both a paracellular and a cholesterol-dependent transcellular mechanism of transendothelial migration during the process of treponemal dissemination.

## Materials and Methods

### Ethics Statements

All animal studies were approved by the local institutional review board at the University of Victoria and were conducted in strict accordance with standard accepted principles as set forth by the Canadian Council on Animal Care (CCAC), National Institutes of Health, and the United States Department of Agriculture in facilities accredited by the American Association for the Accreditation of Laboratory Animal Care and the CCAC. Institutional biosafety approval was obtained under biosafety certificate 13170-010.

### Cloning and Purification of Recombinant Proteins

Treponemal proteins Tp0327 (I23-S172), Tp0751 (V99-P237), and Tp0751 (E115-P237) were cloned as previously described (Houston et al., 2011, 2014; Parker et al., 2016). Tp0327 and Tp0751 constructs were purified from *Escherichia coli* BL21^∗^DE3 and subject to nickel affinity chromatography with HisTrap FF columns (GE Healthcare, Mississauga, ON, Canada) and further purified with size exclusion and cation exchange chromatography (HiLoad 16/60 Superdex 75; GE Healthcare, Chicago, IL, United States) on an AKTA Prime Plus FPLC system (GE Healthcare) in a final buffer of 20 mM HEPES, 150 mM NaCl, 1% glycerol, pH 7.0 as previously described (Houston et al., 2011, 2014; Parker et al., 2016). Labeling of recombinant proteins, Tp0751 (V99-P237) and Tp0327 (I23-S172), with fluorescein isothiocyanate isomer I (FITC I; Sigma-Aldrich, Oakville, Ontario, Canada) was performed as previously described (Lithgow et al., 2020).

### Cloning of SPUD Plasmid

The SPUD gene (Nolan et al., 2006) was commercially synthesized and cloned into the pUC57 vector (GenScript Biotech, Burlington, ON, Canada), and SPUD/pUC57 was transformed into commercial *E. coli* TOP10 cells (Invitrogen, Waltham, MA, United States). Plasmid was isolated using the GeneJET Plasmid Miniprep Kit (Thermo Fisher, Waltham, MA, United States) and DNA concentration was measured by NanoDrop ND-1000 spectrophotometer (Thermo Fisher). Plasmids were diluted to 10^5^ copies/μl in DNase/RNase-free dH_2_O (Thermo Fisher).

### Endothelial Cell Culturing

Primary human umbilical vein endothelial cells (HUVECs, Lonza, Mississauga, ON, Canada) were cultured as previously described (Parker et al., 2016) using VascuLife EnGS Endothelial Medium (Lifeline Cell Technology, Oceanside, CA, United States) devoid of vascular endothelial growth factor. Human brain endothelial cells (hCMEC/d3) were cultured in EndoGro (Millipore, Burlington, MA, United States) supplemented with basic fibroblast growth factor. For recombinant protein or *T. pallidum* traversal assays, Matrigel-coated transwell plates (8 μm pore size, 6.5 mm insert diameter, 24-well plate; Corning) were seeded with HUVECs (3 × 10^4^/well) and grown to confluence (48–72 h) at 37°C in 5% CO_2_. For immunofluorescence assays, eight-well chamber slides (Thermo Fisher Scientific, Burnaby, BC, Canada) were coated with phenol red-free Matrigel (Corning, Tewksbury, MA, United States) by incubating with 500 μg/ml Matrigel for 1 h at room temperature. HUVECs were then seeded at 4 × 10^4^/well and grown for 48 h at 37°C in 5% CO_2_ to form confluent monolayers. For recombinant protein attachment assays or treponemal viability assays, HUVECs or hCMEC/d3 were seeded at 2 × 10^3^/well into 96-well plates (Sarstedt, Sarstedt, Germany) and grown for 24 h at 37°C in 5% CO_2_ to form confluent monolayers. For *T. pallidum* adhesion assays, equal densities of HUVECs were seeded into 24-well tissue culture plates (Corning) to form confluent monolayers for 48 h at 37°C in 5% CO_2_.

### *Treponema pallidum* Propagation

*Treponema pallidum* subsp. *pallidum* (Nichols strain) was propagated in, and extracted from, New Zealand White rabbits as previously described (Lukehart and Marra, 2007). Testicular extractions were performed in 10% normal rabbit serum (NRS) in 0.9% NaCl, and gross cellular debris was removed *via* two centrifugations at 220 × *g* at RT for 5 min. Extractions were conducted at room temperature in an atmosphere of 1.5–5% O_2_ and 5% CO_2_ balanced with N_2_ in a modified anaerobic chamber (Coy Laboratories, Grass Lake, MI, United States). Oxygen levels were monitored with a LabQuest 2 oxygen meter (Vernier, Beaverton, ON, Canada).

### Immunofluorescence Evaluation of VE-Cadherin Junctional Architecture and *T. pallidum* Junctional Localization

For recombinant protein assays, monolayers were treated with gel filtration buffer or equimolar amounts of recombinant Tp0751 (V99-P237) or recombinant Tp0327 (I23-S172) for 30 min at 37°C in 5% CO_2_. Our previous publication confirmed dose-dependent endothelial attachment of recombinant Tp0751 and Tp0327 and demonstrated that 25 μM of recombinant protein results in high levels of Tp0751 binding to endothelial monolayers (Lithgow et al., 2020). An equimolar concentration of Tp0327 (I23-S172) and an equal volume of gel filtration buffer were included in these assays to control for any adverse effects from the presence of recombinant protein or purification buffer. Each well was washed two times with HEPES-buffered saline solution (warmed to room temperature), fixed with 4% paraformaldehyde (PFA) and permeabilized with 0.05% Triton X-100 (TX-100). Immunofluorescence detection of VE-Cadherin and DAPI counterstaining was performed as previously described (Lithgow et al., 2020). For treponemal adhesion assays, *T. pallidum* was isolated from rabbit testicular extractions, quantified using a Petroff-Hausser counting chamber (Hausser Scientific, Horsham, PA, United States) and pre-incubated with anti-Tp0751 serum or NRS (1:2 dilution) for 60 min at 34°C in an atmosphere of 1.5–5% O_2_ as previously described (Lithgow et al., 2020). Anti-Tp0751 was prepared as previously described (Cameron et al., 2005); briefly, New Zealand White rabbits were immunized five times at 3-week intervals with 125 μg of recombinant Tp0751 (54–172) emulsified in the Ribi adjuvant system (Sigma). NRS was purchased from Thermo Fisher Scientific. NRS or anti-Tp0751 serum was heated at 56°C for 30 min to inactivate complement (Lithgow et al., 2020). *T. pallidum* (5 × 10^6^ cells/well) were added to endothelial monolayers and co-incubated for 60 min at 34°C in an atmosphere of 1.5–5% O_2_. The selected inoculum for these studies is based on our previous investigations of *T. pallidum* endothelial adhesion, which demonstrated that an inoculum of 5 × 10^6^ cells/well is sufficient to visualize treponemal attachment to endothelial monolayers (Lithgow et al., 2020). Immunofluorescence detection of VE-cadherin and *T. pallidum* FlaA and DAPI counterstaining and quantification of *T. pallidum* and endothelial cells were performed as previously described (Lithgow et al., 2020). Briefly, samples were examined in a blinded manner using the Cytation 5 software size exclusion settings (BioTek, Nepean, ON, United States). Endothelial nuclei (DAPI; 10–60 μm) and adherent *T. pallidum* (FlaA; 2–8 μm) were quantified in each field of view. For junctional localization of *T. pallidum*, the defined size exclusion settings for *T. pallidum* quantification were applied to generate a mask corresponding to all adherent *T. pallidum* cells in the micrograph (defined as “objects”). For each micrograph, a list of objects (adherent *T. pallidum*, FlaA/TexasRed) was generated that included the mean fluorescence intensity of VE-cadherin from the FITC channel within the mask of the defined object. Adherent treponemal cells (FlaA/TexasRed) were counted as co-distributed with VE-cadherin junctions when the mean fluorescence intensity in the FITC channel (VE-cadherin) for the object exceeded the FITC channel threshold that was automatically determined by the software.

### Endothelial Transwell Assays

#### Transendothelial Electrical Resistance (TEER) Measurements

Endothelial cells cultured in transwell plates were removed from normal growth conditions and incubated at RT for 20 min prior to evaluation of TEER. Barrier integrity was evaluated using an EndOhm chamber with an EVOM-2 voltmeter (World Precision Instruments, Sarasota, FL, United States) to measure resistance, and the TEER was calculated using the following equation:

TEER=REPORTED[R(Ω)TOTAL-R(Ω)BLANK]×M(cm)2AREA

where *TEER*_*REPORTED*_ is the calculated resistance of the barrier in Ω × cm^2^, *R_*TOTAL*_* is the resistance measured across a Matrigel-coated insert seeded with HUVECs, *R*_*BLANK*_ is the resistance measured across a Matrigel-coated insert without cells, and *M*_*AREA*_ is the growth area (0.33 cm^2^) of the transwell insert (Srinivasan et al., 2015). Reported TEER values ≥ 12 Ω × cm^2^ were indicative of an intact endothelial barrier (Dewi et al., 2004).

#### Transwell Solute Flux Assays

Fluorescein isothiocyanate-dextran (40 kDa, 1 mg/ml; Sigma-Aldrich) was added to the apical side of the endothelial monolayers in transwell plates. At time = 0, triplicate 10-μl samples were taken from the upper and lower compartments, and for the remainder of the time course, triplicate 10-μl samples were taken from the lower compartment only. Samples were added to 90 μl of distilled water in a black clear bottom 96-well plate (Corning). Fluorescence of the time-course sample was measured on a BioTek Synergy HT plate reader (BioTek) using a FITC filter (excitation @ 485 nm/emission @ 528 nm) and compared to a standard curve of known concentrations of FITC-dextran to determine the amount of solute present in the lower well. The absolute permeability was calculated with the equation:

P=[C(t)-C(t)0]×VA×t×C0

where *C(t)* is the concentration [μg/ml] of FITC-dextran in the samples that were taken from the lower compartment at each timepoint, *C(t_0_)* is the FITC-dextran concentration [μg/ml] of the samples taken after 0 min, *t* is the duration of the flux (s), *V* is the volume [cm^3^] in the lower compartment, *A* is the surface of the transwell membrane [cm^2^], and *C*_0_ is the initial concentration [μg/ml] of the tracer on the donor side. Results were normalized to the Matrigel-only well lacking endothelial cells (set as 100% flux of the fluorescent tracer). Transwell solute flux assays using FITC-recombinant proteins were performed as described above, but fluorescence was compared to a standard curve of known μg amounts of each FITC-labeled recombinant protein construct to determine the traversal of each recombinant protein.

### Recombinant Protein Barrier Integrity and Traversal Assays

For traversal assays using recombinant protein, cells were treated as indicated with 25 μM of recombinant proteins Tp0751 (V99-P237) or Tp0327 (I23-S172) or FITC-labeled recombinant proteins Tp0751 (V99-P237) or Tp0327 (I23-S172) or control buffer over 290 min.

### *T. pallidum* Endothelial Traversal Assays

For *T. pallidum* assays, transwell inserts of HUVECs were treated with rabbit testicular extract from uninfected rabbits (uninfected TEx, equal volume to *T. pallidum* treatment), 3.2 × 10^7^ live *T. pallidum*, or 3.2 × 10^7^ *T. pallidum* that had been heat-inactivated by incubating at 56°C for 45 min to eliminate treponemal motility (Fitzgerald et al., 1984; Thomas et al., 1988). For traversal inhibition assays, 30 min prior to the addition of *T. pallidum*, endothelial cells in transwell plates were treated with endocytosis inhibitors at concentrations based upon previously published results, including filipin III at 5 μM (Rothberg et al., 1990; Schnitzer et al., 1994; Voigt et al., 2014), 5-(*N*-ethyl-*N*-isopropyl)amiloride (amiloride) at 300 μM (Zhao et al., 2011; Li et al., 2013), (Sigma-Aldrich), Dynasore at 80 μM (Basagiannis et al., 2017; Loh et al., 2017), or Cytochalasin D at 0.1 μM (Wojciak-Stothard et al., 1999). All endocytosis inhibitors were purchased from Sigma-Aldrich. Thirty percent NRS in saline was added to the basal transwell compartments, and suspensions of *T. pallidum* were added to the apical compartment and incubated at 34°C in an atmosphere of 1–3% O_2_ and 5% CO_2_ balanced with N_2_ for 6.5 h or 4 h for darkfield- and qPCR-based traversal assays, respectively. Viability of *T. pallidum* in the apical compartment was monitored by darkfield microscopy at the experimental endpoint (4 or 6.5 h). For darkfield analysis of *T. pallidum* traversal, duplicate samples of 10 μl were collected from each basal compartment at three time points and spotted onto a 1.0–1.2-mm-thick glass slide with a 22 × 36-mm cover glass (Fisher Scientific Company, Ottawa, ON, Canada) and visualized on a Nikon Eclipse E600 darkfield microscope (Nikon Canada, Mississauga, Ontario, Canada) with a ×100 oil immersion objective (×1,000 magnification). For each sample, 10 different fields of view were scanned and the presence of one or more *T. pallidum* cells was recorded as a positive. For qPCR analysis of *T. pallidum* traversal, supernatants were collected from the top and bottom transwell compartments at the assay endpoint (4 h), while inoculum samples were retained for qPCR and processed at the beginning of the experiment. Samples were centrifuged at 14,000 × *g* for 20 min at RT to pellet *T. pallidum*, supernatant was removed, and samples were flash frozen for storage prior to DNA extraction.

### *T. pallidum* Adhesion, Association, and Viability Assays

Human umbilical vein endothelial cells were seeded in six-well plates (adhesion assay), transwell plates (association), or 96-well plates (viability assays) coated with Matrigel (Corning) and grown to confluence. Confluent monolayers were incubated with 5 μM filipin or no inhibitor (control) in culture medium for 30 min at 37°C/5% CO_2_. All further manipulations were performed in microaerophilic conditions (1–3% O_2_, 5% CO_2_ balanced with N_2_). For adhesion assays, HUVECs were incubated with *T. pallidum* (1 × 10^6^ cells/well) for 1 h. Monolayers were washed three times with 10% NRS in normal saline. Endothelial cells in six-well plates (“adhesion assays”) or the endothelial cell layers from transwell assays (“association assays”) were lysed by addition of 200 μl lysis buffer (10 mM Tris–HCl pH 8.0, 0.1 M EDTA, and 0.5% SDS in DNase/RNase-free dH_2_O; Thermo Fisher). Lysates were incubated with 50 μl proteinase K (Qiagen, Toronto, ON, Canada) at 56°C for 10 min, then flash frozen for storage prior to DNA extraction. For *T. pallidum* viability assays, HUVEC monolayers were exposed to filipin (5 μM) for 30 min at 37°C/5% CO2. After inhibitor exposure, *T. pallidum* (2 × 10^7^ cells/well) were added in triplicate to filipin-treated or untreated HUVEC monolayers. Treponemal motility was evaluated in a blinded manner at four different time points (0, 90, 180, and 240 min) by taking 10-μl samples from the coculture supernatants and observing five fields of view, or a maximum of 200 *T. pallidum*, to assess motility. At the experimental endpoint (*T* = 270 min), HUVECs and *T. pallidum* were dissociated from plates using the protocol previously described for subculture of *T. pallidum* and Sf1Ep NBL-11 cottontail rabbit epithelial cells during long-term *in vitro* cultivation (Edmondson et al., 2018). Briefly, HUVECs and *T. pallidum* were incubated with 0.25% trypsin-EDTA (Sigma) for 5 min at 37°C in 5% CO2 and treponemal motility was evaluated from dissociated samples.

### *Treponema pallidum* Genomic DNA (gDNA) Extraction

For traversal assays, frozen pellets were resuspended in 200 μl of lysis buffer of 10 mM Tris–HCl, pH 8.0 0.1 M EDTA, and 0.5% SDS in DNase/RNase-free dH_2_O (Thermo Fisher). As an extraction efficiency control, 10^6^ copies of SPUD plasmid were spiked into the lysate. Samples were then incubated with buffer AL and proteinase K (DNeasy Blood and Tissue Kit, Qiagen), vortexed for 10 s, and incubated at 56°C for 10 min; 200 μl of 95% ethanol was added to the mixture and vortexed before loading onto spin columns. gDNA was extracted with the DNeasy blood and tissue kit (Qiagen) as per the manufacturer’s instructions. Samples were eluted in 20 μl RNAse-free DNAse-free PCR-grade distilled water (Thermo Fisher Scientific). For samples from six-well plates (adhesion assays) or endothelial layers from transwell plates (association assays), lysates were incubated at 70°C for 10 min to inactivate proteinase K. Samples were then mixed with 200 μl Buffer AL, 10^6^ copies SPUD plasmid, and 200 μl 95% EtOH before loading on to spin columns. DNA was eluted in 30 μl DNase/RNase-free dH_2_O (Thermo Fisher).

### Quantitative Real-time PCR

Quantitative real-time PCR (qPCR) was performed on gDNA extractions of *T. pallidum* from transwell plate assays or adhesion assays using SsoFast^TM^ EvaGreen^®^ Supermix (Bio-Rad, Mississauga, ON, Canada). As previously described (Lithgow et al., 2017; Lithgow et al., 2020), quantification of *T. pallidum* gDNA was determined using HPLC-purified primers (Integrated DNA Technologies, Coralville, IA, United States) targeting the endoflagellar sheath protein (*flaA*) gene (GenBank accession number M63142.1). The sense primer (5′-AACGCAAACGCAATGATAAA-3′) anneals to bases 475 to 494, and the antisense primer (5′-CCAGGAGTCGAACAGGA GATAC-3′) anneals to bases 738 to 759 of *flaA*. Quantification of HUVEC gDNA was determined using HPLC-purified primers (Integrated DNA Technologies) targeting exon 6 of the glyceraldehyde-3-phosphate dehydrogenase (*GAPDH*) gene (GenBank accession number NG_007073). The sense primer (5′-AGCAAGAGCACAAGAGGAAGAG-3′) anneals to bases 3676 to 3697, and the antisense primer (5′-GAGCACAGGGTACTTTATTGATGG-3′) anneals to bases 3827 to 3850. A *GAPDH* standard curve was generated using a two-fold serial dilution of HUVEC gDNA from 84.1 to 0.041 ng/μl with an efficiency of 98.4% and an *R*^2^ value of 0.981 (Lithgow et al., 2020). As previously described, *Solanum tuberosum* phyB gene (GenBank Y14572) or ‘SPUD,’ encodes a species-specific gene that can be spiked into experimental samples. SPUD has previously been used as an internal amplification control (Nolan et al., 2006). In this study, SPUD plasmid DNA was spiked into samples before DNA extraction to normalize DNA extraction efficiency across samples and experiments. HPLC-purified primers (Integrated DNA Technologies) targeting SPUD included the sense primer (5′-AACTTGGCTTTAATGGACCTCCA-3′) and antisense primer (5′-ACATTCATCCTTACATGGCACCA-3′) (Nolan et al., 2006); SPUD standard curves were generated by 10-fold serial dilution of SPUD plasmid from 10^7^ to 10^2^ copies/μl. All reactions (20 μl) were performed in triplicate with SsoFast^TM^ EvaGreen^®^ Supermix, 500 nM primers, and 1 μl of template (extracted gDNA). Assays were run on a Bio-Rad CFX Connect Real-Time PCR Detection System (Bio-Rad Laboratories, Mississauga, ON, Canada) using twin.tec^TM^ skirted 96-well plates (Eppendorf, Mississauga, ON, Canada) sealed with microseal B film (Bio-Rad Laboratories). PCR conditions were as follows: 95°C for 2 min, followed by 40 amplification cycles of 95°C for 10 s, and 15 s at the appropriate annealing temperature (*GAPDH:* 60°C, *flaA:* 65°C, *SPUD*: 65°C). Following 40 cycles, there was a final denaturation step for 10 s at 95°C and melt curve analysis from 65 to 95°C in 0.5°C increments for 5 s each (*flaA, SPUD, GAPDH*). Each assay was run with the following controls: negative controls including no template control and no primer control, and positive control including linearized *flaA* plasmid as the template. Data was analyzed using Bio-Rad Maestro software, version 4.1.2. Standard curves produced for *GAPDH*, *flaA*, and *SPUD* were used to determine sample DNA concentrations, and concentrations were normalized within biological replicates. For traversal assays, the SPUD normalization factor was determined by dividing the calculated SPUD copies measured in the extraction by the expected SPUD copies (10^6^/extraction). The calculated *flaA* copies per extraction were divided by the SPUD normalization factor to account for differences in extraction efficiency between experiments and replicates. For adhesion and association assays, calculated *flaA* copies measured in the extraction were divided by GAPDH copies as previously described (Lithgow et al., 2020).

### Endothelial Cell Attachment Assays Using Recombinant Treponemal Proteins

As previously described (Lithgow et al., 2020), equal densities of endothelial cells were seeded in 96-well plates (Sarstedt). For inhibitor assays, monolayers were incubated with filipin (1.5, 4.5, or 7.5 μM in culture medium) for 30 min at 37°C/5% CO_2_ prior to the addition of recombinant protein. Endothelial cells were incubated with 15 μM of recombinant treponemal proteins: Tp0751 (E115-P237), Tp0751 (V99-P237), Tp0327 (I23-S172), or FITC-labeled Tp0751 (V99-P237) for 90 min at 37°C in an atmosphere of 5% CO_2_, then monolayers were gently washed three times with warm HBSS to remove non-adherent protein, fixed in 2% PFA, and blocked for 30 min in 1% BSA in PBS at 37°C in an atmosphere of 5% CO_2_. Attachment of recombinant proteins to endothelial monolayers was evaluated by detecting the N-terminal hexa-histidine tags on recombinant proteins using nickel-labeled horseradish peroxidase (Ni-HRP; Mandel Scientific, Guelph, ON, Canada), and plates were developed with a 3,3′,5,5′-tetramethylbenzidine (TMB) substrate system (Mandel Scientific) and read at OD600 nm on a BioTek Synergy HT (BioTek, Nepean, ON, Canada).

### Endothelial Viability and Cell Death Assays

After inhibitor exposure, HUVEC cell viability was assessed with the CellTiter-Glo^®^ assay (Promega, Madison, WI, United States) and cytotoxicity was evaluated using the CellTox Green^®^ Assay (Promega). Endothelial cells seeded at 2 × 10^3^/well in black clear bottom 96-well plates (Corning) were exposed to different concentrations of endocytosis inhibitors for 30 min at 37°C/5% CO_2_. For cell viability assays, media were added to each well after inhibitor incubations and the plate was equilibrated to room temperature. CellTiter-Glo^®^ Reagent was added to each well, and luminescence was read on a BioTek Synergy HT (BioTek). For cytotoxicity assays, CellTox Green^®^ Reagent was added to each well after inhibitor treatments and incubated for 15 min at room temperature. Fluorescence was measured using a FITC filter (excitation @ 485 nm/emission @ 528 nm) on a BioTek Synergy HT (BioTek).

### Microscopy and Cell Counting

Micrographs were captured with a Nikon Eclipse E600 darkfield microscope (Nikon Canada, Mississauga, Ontario, Canada) with a ×40 objective (×400 magnification) or a Cytation 5 Imaging Reader (BioTek) with a ×20 objective (×200 magnification). As previously described (Lithgow et al., 2020), for *T. pallidum* adhesion assays, samples were blinded and the numbers of endothelial nuclei (DAPI; 10–60 μm) and *T. pallidum* cells (FlaA; 2–8 μm) were measured from each field of view with the Cytation 5 software using size exclusion quantification settings (BioTek). Mean fluorescence intensity of VE-cadherin was quantified using the Cytation 5 Imaging Reader (BioTek) with a ×20 objective at five random fields of view per well.

### Statistical Analysis

Statistical analyses were performed in GraphPad Prism (GraphPad) or R (R Core Team, 2017) as denoted in figure legends. Data normality was analyzed using the Shapiro–Wilk test (GraphPad). Graphs were prepared in GraphPad Prism (GraphPad) or Excel (Microsoft).

## Results

### *Treponema pallidum* Localizes to Intercellular Adherens Junctions

**FIGURE 1:**
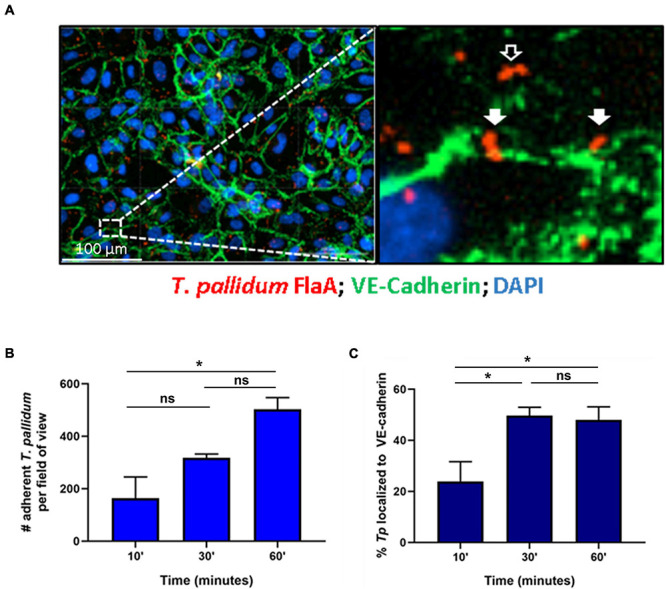
*Treponema pallidum* localizes to endothelial intercellular junctions. **(A)** Representative immunofluorescence image of *T. pallidum* attached to endothelial cells. Inset reveals *T. pallidum* attached to endothelial cells in non-junctional regions (black arrow) and localized to VE-cadherin (white arrows). *T. pallidum* was co-incubated with confluent HUVEC monolayers for 10, 30, or 60 min. After washing to remove non-adherent treponemes, *T. pallidum* and HUVECs were fixed and permeabilized and *T. pallidum* attachment to endothelial monolayers was assessed *via* immunofluorescent staining of the *T. pallidum* periplasmic flagellar protein (FlaA, red) for treponemal quantification; HUVEC nuclei (DAPI, blue) and cellular margins (VE-cadherin, green) were stained to evaluate *T. pallidum* localization to cellular margins (green) or non-junctional areas. Colocalization with adherens junctions was evaluated by quantifying *T. pallidum* attached at sites with overlapping signal between FlaA (red; *T. pallidum*) and intercellular junctions (green; VE-cadherin). **(B)** Time course of *T. pallidum* attachment to HUVECs after (*i*) 10-min (*ii*) 30-min, and (*iii*) 60-min co-incubations. Statistical significance was evaluated by one-way ANOVA comparing *T. pallidum* attachment over the time course (*p* = 0.048) with Tukey’s *post hoc* comparison evaluating *T. pallidum* attachment between 10- and 30-min time points (*p* = 0.25), 10- and 60-min time points (*p = 0.0427), and 30- and 60-min time points (ns; *p* = 0.1803). **(C)** Percent localization of *T. pallidum* to VE-cadherin intercellular junctions. Results are presented as mean ± SEM from three independent experiments. Statistical significance was evaluated by one-way ANOVA with Tukey’s *post hoc* comparison evaluating *T. pallidum* localization to junctions between 10- and 30-min time points (*p = 0.0401), 10- and 60-min time points (*p = 0.0482), and 30- and 60-min time points (ns; *p* = 0.9503). Images were acquired at ×200 magnification using a Cytation 5 Imaging Reader.

*Treponema pallidum* localization to intercellular junctional regions of endothelial cells has been previously reported (Thomas et al., 1988). To further explore this tropism for endothelial junctions, a time-course evaluation of *T. pallidum* attachment to human umbilical vein endothelial cells (HUVECs) was conducted and treponemal localization to areas overlapping with the intercellular adherens junction protein VE-cadherin, a key structural and regulatory molecule of vascular barriers (Dejana et al., 2008), was evaluated (Figure 1). Over a 60-min time course, *T. pallidum* exhibited increased attachment to HUVEC monolayers (Figure 1B; *p* = 0.048). Junctional localization was observed in 23.9% of the treponemal population after a 10-min *T. pallidum*-endothelial co-incubation (Figure 1C), and there was a significant increase in junctional localization for subsequent 30-min and 60-min co-incubations (Figure 1C; *p* = 0.0401, *p* = 0.0482). After 30-min of *T. pallidum*-HUVEC co-incubations, junctional localization reached a plateau in which half of the treponemal population was localized to intercellular junctions (Figure 1C). Localization to VE-cadherin was determined based on overlapping pixel intensity between *T. pallidum* FlaA and VE-cadherin (Figure 1A; inset, white arrows) and compared to *T. pallidum* attachment in non-junctional regions (Figure 1A; inset, black arrows).

### *Treponema pallidum* Modifies Endothelial Junctional Architecture of VE-Cadherin

**FIGURE 2:**
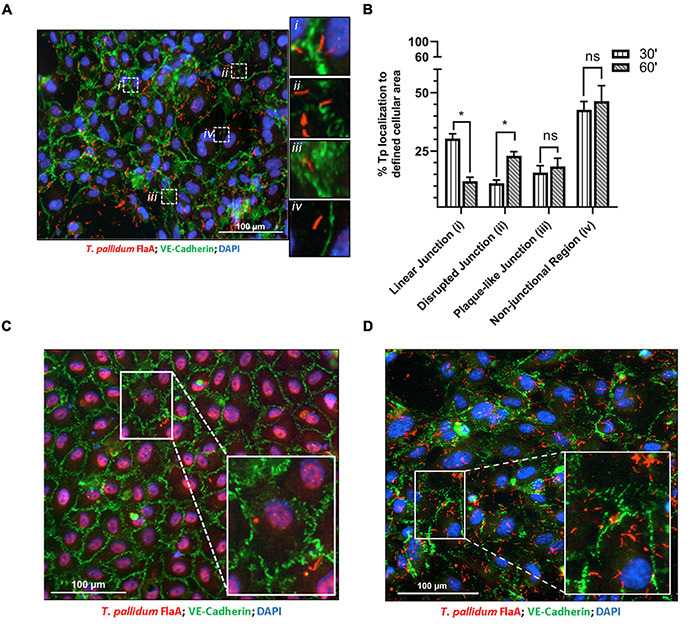
*Treponema pallidum* modifies endothelial VE-cadherin architecture. **(A)** Immunofluorescence image of *T. pallidum* attached to a HUVEC monolayer after a 60-min co-incubation. Immunofluorescence of FlaA (*T. pallidum;* red), DAPI (nuclear stain, blue), and intercellular junctions (VE-cadherin, green) shown at ×200 magnification obtained with a Cytation 5 Imaging Reader. Insets highlight *T. pallidum* localized to defined regions including (*i*) linear (undisrupted) junctions, (*ii*) disrupted junctions, (*iii*) plaque-like junctions, and (*iv*) non-junctional areas. **(B)** Percent localization of *T. pallidum* to defined cellular regions at 30- and 60-min time points. Results presented as mean ± SEM from three independent experiments; statistical significance was assessed with a two-way ANOVA and Sidak’s multiple-comparison test evaluating percent localization to cellular structures between 30- and 60-min time points for linear junction (*p = 0.032), disrupted junction (*p = 0.048), plaque-like junction (ns *p* = 0.91), and non-junctional area (ns *p* = 0.99). **(C,D)** Immunofluorescence images of *T. pallidum* attached to HUVECs following treponemal pre-incubation with **(C)** Tp0751-specific serum and **(D)** normal rabbit serum. Representative images at ×200 from three independent experiments. Insets highlight *T. pallidum* attached to endothelial cells with **(C)** intact junctions in samples treated with Tp0751-specific serum and **(D)** disrupted junctions in samples treated with normal rabbit serum.

Previous investigations have characterized morphologies of endothelial intercellular junctions that include (1) continuous junctions where VE-cadherin is observed in linear arrangement between cells; (2) interrupted junctions with a punctate or discontinuous pattern of VE-cadherin; (3) adhesion plaques where VE-cadherin localizes to the plasma membrane during an intermediate overlap of plasma membrane between cells; and (4) non-junctional VE-cadherin which includes junctional protein internalization (Abu Taha and Schnittler, 2014; Seebach et al., 2015, 2016; Cao et al., 2017; Cao and Schnittler, 2019). To evaluate whether *T. pallidum* junctional localization results in modification of VE-cadherin architecture, adherent *T. pallidum* were grouped into these four categories based on localization of bacteria (Figure 2A *i-iv*, respectively) and compared between 30- and 60-min time points. Since *T. pallidum* localization to VE-cadherin junctions did not differ between these two time points (Figure 1C), this analysis explored whether the junctional architecture in regions of *T. pallidum* adhesion changed between these two time points. Consistent with the junctional co-localization analysis (Figure 1C), there was no change in the abundance of *T. pallidum* localized to non-junctional regions (Figure 2A inset *iv*) at 30- and 60-min time points (Figure 2B, *iv*, ns *p* = 0.64). Similarly, *T. pallidum* localization to plaque-like junctional regions (Figure 2A inset *iii*) did not differ between the two time points (Figure 2B, *iii*, ns *p* = 0.60). *T. pallidum* colocalization with linear junctions (Figure 2A inset *i*) decreased from 30.4 to 12.1% between 30- and 60-min time points (Figure 2B ^∗^*p* = 0.04), while the opposite effect was observed for *T. pallidum* in areas of junctional disruption (Figure 2A inset *ii*) where localization increased from 11.2% at 30-min to 23.0% at 60-min (Figure 2B, *ii*, ^∗∗^*p* = 0.002). This shift from a higher proportion of *T. pallidum* observed within linear junctions at 30-min to a higher proportion of *T. pallidum* observed within areas of junctional disruption at 60-min (Figure 2B and Supplementary Figure 1) is consistent with *T. pallidum* contributing to junctional disruption of VE-cadherin. Previous work has shown that serum specific for the *T. pallidum* vascular adhesin Tp0751 blocks *T. pallidum* adhesion (Lithgow et al., 2020). When treponemal cells were incubated with Tp0751-specific serum prior to the HUVEC co-incubation, no areas of junctional disruption were observed (Figure 2C), demonstrating that *T. pallidum* attachment to endothelial monolayers is required to promote changes in VE-cadherin architecture. Conversely, no such inhibition of junctional disruption was observed for *T. pallidum* pre-incubated with NRS (Figure 2D). This finding also reveals that host factors present in the *T. pallidum in vivo* propagation extraction mixture are not driving junctional modification in HUVECs, as junctional changes are only observed in samples with adherent treponemes.

### Tp0751 Modifies the Architecture of Endothelial Adherens Junctions

**FIGURE 3:**
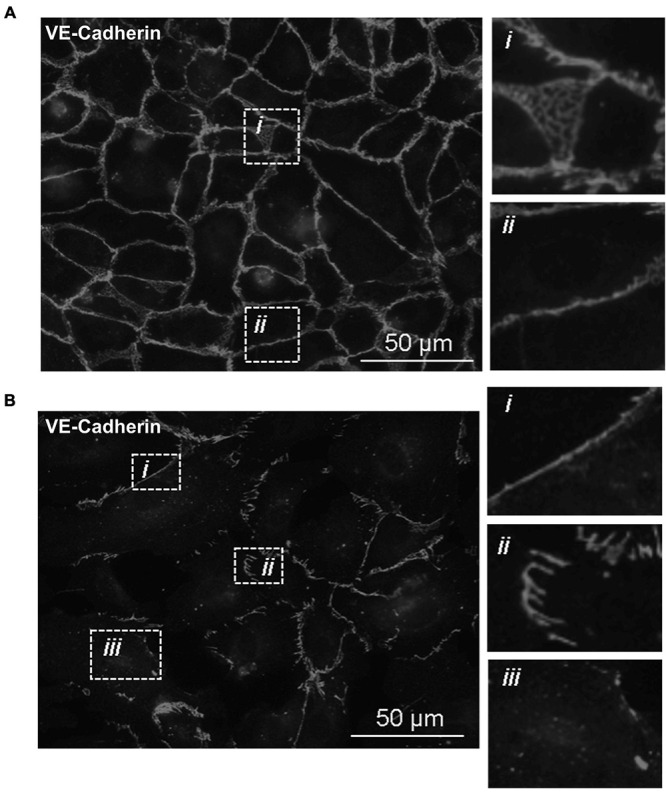
Recombinant Tp0751 disrupts endothelial VE-cadherin intercellular junctions. Immunofluorescence evaluation of the distribution of the adherens junction protein VE-cadherin in human umbilical vein endothelial cells (HUVECs) following a 30-min co-incubation with 25 μM of recombinant protein. Shown are representative immunofluorescence images of permeabilized endothelial cells stained with mouse anti-VE-cadherin and goat anti-mouse (Alexa 568-conjugated) from three independent experiments obtained from a Nikon 80i fluorescence microscope at ×400 magnification. Insets reveal specific junctional architecture. **(A)** HUVECs treated with negative control recombinant Tp0327 (I23-S172) where insets reveal (*i*) VE-cadherin plaque-like junctional structure and (*ii*) linear (continuous) junctions. **(B)** HUVECs treated with recombinant Tp0751 (V99-P237) where insets reveal (*i*) linear (continuous) junctions, (*ii*) disrupted (discontinuous) junctions, and *(iii*) intracellular VE-cadherin.

**FIGURE 4:**
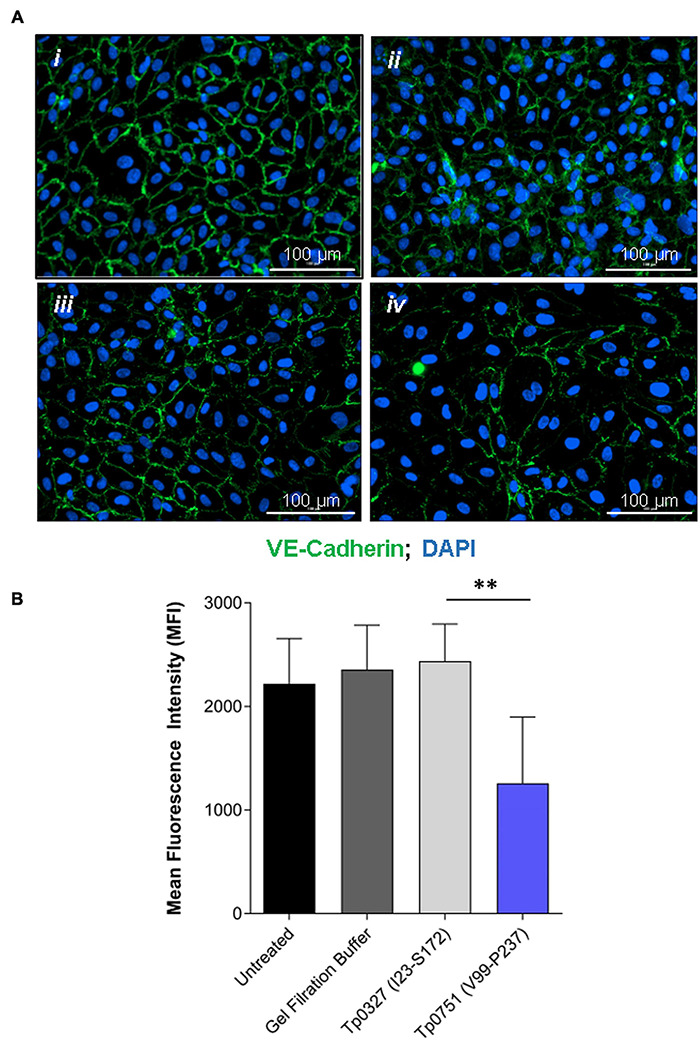
Junctional VE-cadherin is reduced in endothelial cells after exposure to Tp0751. **(A)** Representative immunofluorescence images from HUVECs treated with *(i)* media only, *(ii)* gel filtration buffer, *(iii)* 30 μM negative control recombinant Tp0327 (I23-S172), or *(iv)* 30 μM recombinant Tp0751 (V99-P237) obtained using a Cytation 5 Imaging Reader at ×200 magnification. HUVEC nuclei were stained with DAPI (blue), and cellular margins were stained with mouse monoclonal anti-VE-cadherin followed by goat anti-mouse (Alexa 488-conjugated, green). HUVEC membranes remained intact (non-permeabilized) to eliminate intracellular pools of VE-cadherin from the analysis. **(B)** Quantification of junctional architecture in HUVECs using mean fluorescence intensity (MFI) of VE-cadherin per field of view after treatment with media only, gel filtration buffer, negative control recombinant Tp0327 (I23-S172), or recombinant Tp0751 (V99-P237). Results presented as MFI ± standard error of the mean (SEM) from five random fields of view per well performed in technical duplicate from three independent experiments. Statistical significance was evaluated using Student’s *t*-test comparing Tp0751-treated endothelial monolayers to Tp0327-treated endothelial monolayers (**p = 0.007).

With the knowledge that Tp0751 functions as a *T. pallidum* vascular adhesin (Parker et al., 2016; Kao et al., 2017; Lithgow et al., 2020), these investigations sought to understand whether Tp0751 could elicit functional changes in endothelial monolayers that mirror the effects seen with *T. pallidum*. To this end, the architecture of VE-cadherin was evaluated in HUVECs following exposure to recombinant Tp0751 or a negative control recombinant protein, Tp0327. Using the four classifications described above, we determined that endothelial monolayers treated with the negative control recombinant protein, Tp0327, retained linear VE-cadherin junctional patterns (Figure 3A; inset *ii*) with some VE-cadherin plaques (Figure 3A; inset *i*), while Tp0751-treated monolayers primarily exhibited discontinuous junctions (Figure 3B; inset *ii*) and intracellular VE-cadherin (Figure 3B; inset *iii*) with some instances of linear VE-cadherin junctional morphology (Figure 3B; inset *i*). To further explore Tp0751-mediated disruption of adherens junction architecture, a quantitative immunofluorescence microscopy approach was used to analyze changes in the mean fluorescence intensity per field of view of VE-cadherin in HUVEC monolayers following exposure to recombinant proteins and controls. Monolayers were not subjected to permeabilization to ensure specific immunofluorescence quantification of VE-cadherin at intercellular junctions, and to exclude intracellular pools of internalized junctional protein. This analysis revealed that Tp0751-treated monolayers had a reduction in cell-surface junctional VE-cadherin when compared to the controls: untreated, gel-filtration buffer-treated, or negative control recombinant Tp0327-treated monolayers (Figure 4). Statistical significance was assessed by comparing Tp0751-treated monolayers with the appropriate negative control, Tp0327-treated monolayers (Figure 4B; ^∗∗^*p* = 0.007).

### Recombinant Tp0751 Does Not Alter the Permeability of Endothelial Barriers

**FIGURE 5:**
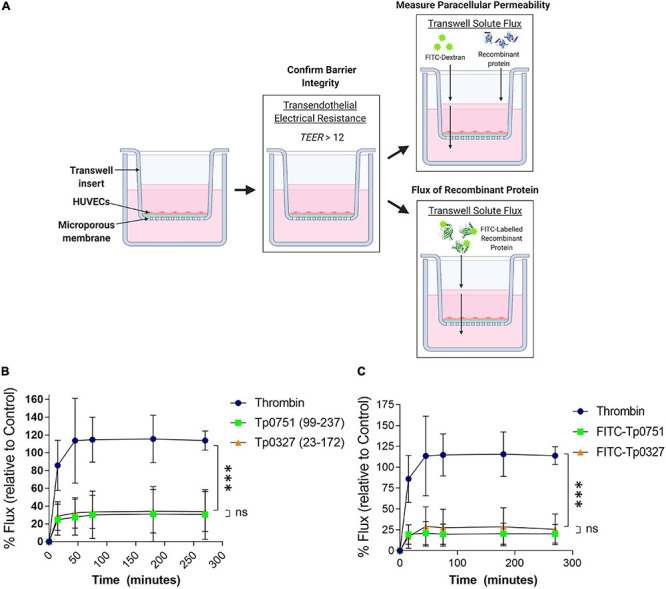
Tp0751 does not alter barrier integrity or traverse endothelial barriers. **(A)** Schematic of transwell solute flux assay of endothelial monolayers exposed to recombinant treponemal proteins. HUVECs were seeded onto an artificial basement membrane in transwell inserts and grown to confluence. Barrier integrity was confirmed using TEER, where monolayer resistance > 12Ω × cm^2^ indicated an intact monolayer. Throughout the time course of the experiments, paracellular permeability was assessed using a transwell solute flux assay, where endothelial permeability in the presence of recombinant treponemal proteins was monitored with a FITC-dextran fluorescent tracer by sampling from the bottom compartment of the transwell for fluorescence measurements. Recombinant protein traversal was evaluated by monitoring the flux of FITC-labeled recombinant proteins across the monolayer. Image created with BioRender. **(B)** Solute permeability of HUVEC monolayers was evaluated with the fluorescent tracer FITC-dextran in the presence of recombinant Tp0751 (V99-P237) and negative control recombinant Tp0327 (I23-S172) and compared to the vasoactive agent thrombin (positive control) over 4.5 h. Results are normalized to transwells containing artificial basement membrane without endothelial cells (set as 100% flux of FITC-dextran) and presented as mean ± SEM from three independent experiments. Statistical significance was assessed by two-way ANOVA ns *p* = 0.559 [time factor], ^∗^*p* = 0.0212 [treatment factor], with Tukey’s posttest, thrombin vs. Tp0751 ^∗∗∗^*p* = 0.0003; thrombin vs. Tp0327 ^∗∗∗^*p* = 0.0002, Tp0751 vs. Tp0327 ns *p* = 0.720. **(C)** Solute permeability of HUVEC monolayers and recombinant protein traversal was evaluated by monitoring the flux of FITC-Tp0751 and FITC-Tp0327 (negative control) across the monolayer, compared to flux of FITC-dextran across monolayers in the presence of the vasoactive agent thrombin (positive control) over 4.5 h. Results are normalized to transwells containing artificial basement membrane without endothelial cells (set as 100% flux of FITC-dextran) and presented as mean ± SEM from three independent experiments. Statistical analyses were performed using a two-way ANOVA ns *p* = 0.577 [time factor], ^∗^*p* = 0.0277 [treatment factor] with Tukey’s posttest, thrombin vs. Tp0751 ^****^p < 0.0001; thrombin vs. Tp0327 ^****^p < 0.0001, Tp0751 vs. Tp0327 ns *p* = 0.090.

To assess the effect of Tp0751-mediated junctional remodeling on endothelial permeability, solute flux was measured across endothelial monolayers after Tp0751 exposure. HUVECs were seeded onto transwell plates coated with an artificial basement membrane, and TEER measurements were performed at the start of each experiment to confirm barrier integrity (Figure 5A). To monitor barrier integrity during the experiment, flux of the fluorescent tracer, fluorescein isothiocyanate-dextran (FITC-dextran), across the endothelial barrier was monitored by measuring fluorescence in the bottom compartment of the transwells throughout the time course (Figure 5A). Monolayers were incubated with recombinant Tp0751, recombinant Tp0327 (negative control), or thrombin (positive control), a known vasoactive agent that increases the permeability of endothelial barriers (Gavard, 2013). Flux of the fluorescent tracer, fluorescein isothiocyanate-dextran (FITC-dextran), across the endothelial barrier was monitored over the time course of the experiment (Figure 5A). Endothelial monolayers exposed to the positive control thrombin exhibited elevated permeability within the first 50-min compared to monolayers exposed to either recombinant protein (Figure 5B; thrombin vs. Tp0751 ^∗∗∗^*p* = 0.0003; or vs. Tp0327 ^∗∗∗^*p* = 0.0002). Conversely, Tp0751 did not elicit any change in endothelial barrier permeability when compared to monolayers exposed to the negative control recombinant protein Tp0327 (Figure 5B; ns Tp0751 vs. Tp0327 *p* = 0.720), demonstrating that recombinant Tp0751 does not affect barrier integrity of HUVEC monolayers at the time points measured.

### Recombinant Tp0751 Does Not Traverse Endothelial Barriers

Since recombinant Tp0751 does not impact the paracellular permeability of endothelial monolayers (Figure 5B), the capability for recombinant Tp0751 to traverse monolayers through paracellular or transcellular routes independent of barrier permeability alteration was explored. Recombinant Tp0327 and recombinant Tp0751 were chemically labeled with FITC to allow for fluorescent tracking of recombinant protein traversal across the monolayers (Figure 5A). Plate-based endothelial binding assays confirmed that FITC labeling did not affect the cell-binding capacity of Tp0751 (Supplementary Figure 2; ns *p* = 0.116). Confluent monolayers were co-incubated with FITC-labeled recombinant protein and protein flux into the bottom compartment was monitored throughout the 4.5-h time course (Figure 5A). Neither FITC-Tp0751 nor FITC-Tp0327 traversed across the endothelial barrier (Figure 5C; ns *p* = 0.499), while thrombin still increased barrier permeability as demonstrated by increased flux of FITC-dextran across the monolayer (Figure 5C).

### *Treponema pallidum* Traverses Endothelial Monolayers Without Disrupting Barrier Permeability

**FIGURE 6:**
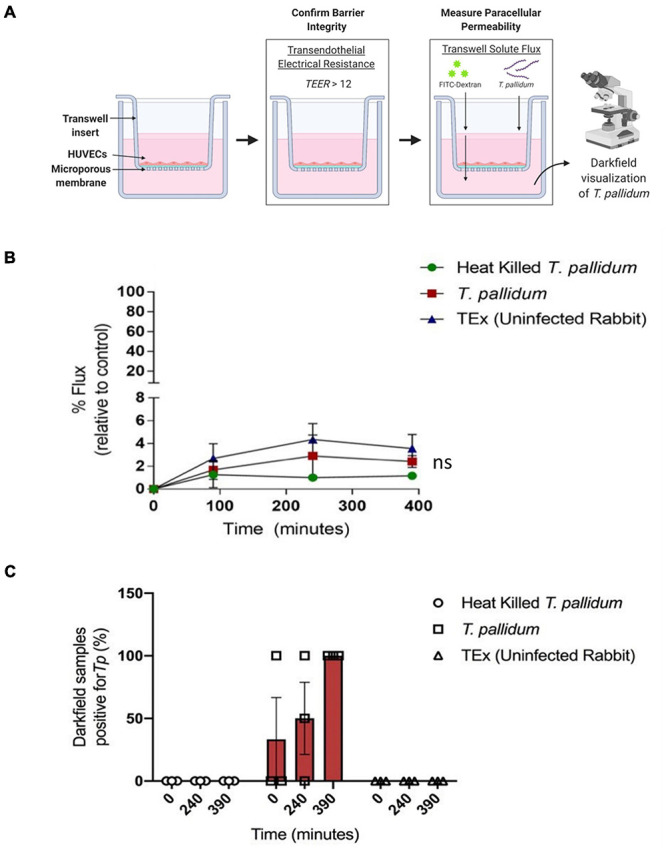
*Treponema pallidum* traverses endothelial barriers without disrupting barrier integrity. **(A)** Schematic of transwell solute flux assay of endothelial monolayers exposed to *T. pallidum*. HUVECs were seeded onto an artificial basement membrane in transwell inserts and grown to confluence. Barrier integrity was confirmed using TEER, where monolayer resistance > 12Ω × cm^2^ dictated an intact monolayer. Barrier permeability was assessed with a transwell solute flux assay, where endothelial permeability in the presence of *T. pallidum* was monitored with a FITC-dextran fluorescent tracer by sampling from the bottom compartment of the transwell for fluorescence measurements throughout the time course. Image created with BioRender. **(B)** Solute permeability of HUVEC monolayers was evaluated with the fluorescent tracer FITC-dextran in the presence of *T. pallidum*, heat-killed *T. pallidum*, or testicular extract from uninfected rabbits over 6.5 h. Results are normalized to transwells containing artificial basement membrane without endothelial cells (set as 100% flux of FITC-dextran) and presented as mean ± SEM from three independent experiments. Statistical significance was analyzed by two-way ANOVA comparing *T. pallidum*, heat-killed *T. pallidum*, and testicular extract from uninfected rabbits [“TEx (Uninfected Rabbit)”]; treatment factor ns *p* = 0.707; time factor *p* = 0.004. **(C)** Darkfield analysis of duplicate 10-μl samples taken from the bottom chambers from endothelial cells treated with live *T. pallidum*, heat-killed *T. pallidum*, or TEx (Uninfected Rabbit) from three separate experiments at each timepoint. Data points represent the percentage of darkfield positive samples from each independent experiment. Slides were scanned for 10 different fields of view and the presence of ≥ 1 *T. pallidum* cell was recorded as a positive.

**FIGURE 7:**
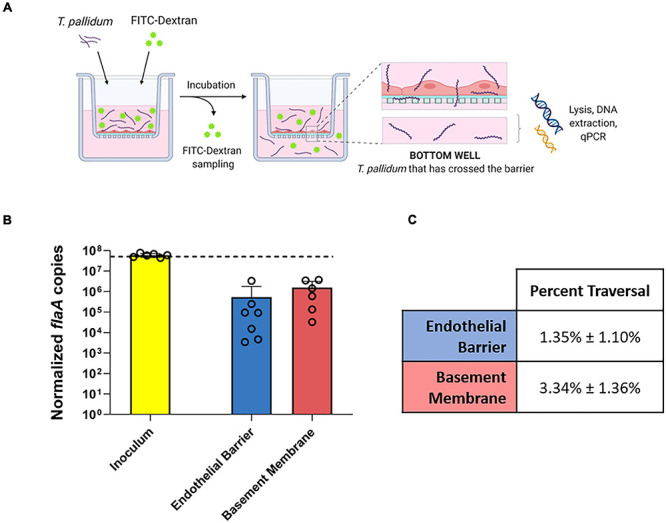
Quantification of *T. pallidum* traversal across endothelial barriers and basement membranes. **(A)** Schematic of *T. pallidum* traversal assay. HUVECs were seeded onto an artificial basement membrane in transwell inserts and grown to confluence. Barrier integrity was confirmed using transendothelial electrical resistance (TEER), where monolayer resistance > 12Ω × cm^2^ dictated an intact monolayer. Barrier permeability was assessed with a transwell solute flux assay, where endothelial permeability in the presence of *T. pallidum* was monitored with a FITC-dextran fluorescent tracer by sampling from the bottom compartment of the transwell for fluorescence measurements throughout the 4-h period. *T. pallidum* traversal was evaluated by collecting the media from the lower compartment in each well and quantifying traversal with quantitative polymerase chain reaction (qPCR) detection of endoflagellar sheath gene *flaA*. Image created with BioRender. **(B)** Starting inoculum of *T. pallidum* (yellow) and *T. pallidum* traversed across endothelial barriers (blue) or basement membranes (red) quantified with qPCR targeting the endoflagellar sheath gene, *flaA*, normalized to a spiked-in plasmid DNA to control for differences in extraction efficiency. Results presented as mean ± SEM from six independent experiments. **(C)** Percent traversal of *T. pallidum* across endothelial barriers or basement membranes relative to the starting inoculum.

To explore the functional consequence of *T. pallidum* junctional remodeling and investigate the mechanisms of *T. pallidum* transendothelial migration, barrier permeability was monitored during *T. pallidum* traversal across endothelial monolayers. Paracellular permeability was evaluated *via* transwell solute flux of FITC-dextran across endothelial monolayers during exposure to live *T. pallidum*, heat-killed *T. pallidum*, or testicular extract from uninfected rabbits (Figure 6A). While darkfield microscopic analysis of samples from the bottom chamber revealed that live *T. pallidum* traversed endothelial barriers (Figure 6C), no significant changes in barrier permeability were observed in endothelial monolayers exposed to any of the treatments over the 390-min (6.5-h) time course (Figure 6B; ns *p* = 0.707 [treatment factor]). To more accurately quantify *T. pallidum* transendothelial migration, a quantitative PCR (qPCR)-based method was established to determine the number of *T. pallidum* cells in the starting inoculum and in the lower compartment of wells containing HUVECs seeded onto artificial basement membranes or artificial basement membranes alone (Figure 7A). To control for differences in DNA extraction efficiency, *flaA* quantification was normalized to plasmid DNA that was spiked in prior to extraction (SPUD plasmid, 10^6^ copies). As expected, *Treponema pallidum* displayed increased migration across basement membranes alone compared to endothelial barriers seeded onto basement membranes (Figure 7B). A traversal rate of 1.35% ± 1.10 was observed for *T. pallidum* migration across endothelial barriers, while the *T. pallidum* traversal rate across artificial basement membranes was 3.34% ± 1.36 (Figures 7B,C).

### *Treponema pallidum* Transendothelial Migration Is Reduced With a Lipid Raft/Caveolae-Mediated Endocytosis Inhibitor

**FIGURE 8:**
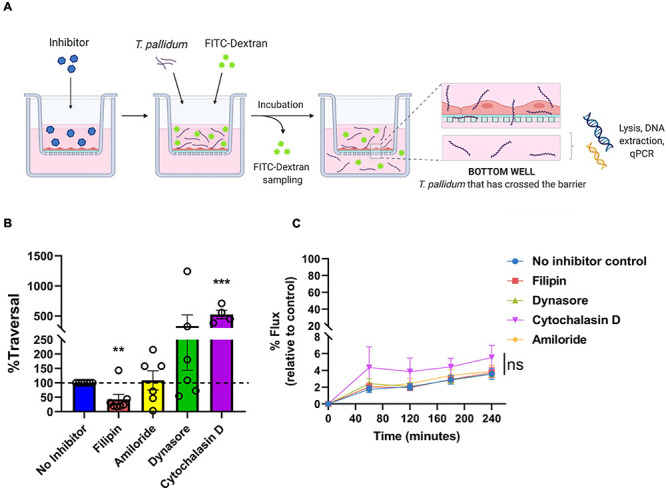
*Treponema pallidum* traversal of endothelial barriers is partially abrogated with an inhibitor of lipid raft-mediated endocytosis. **(A)** Schematic of transendothelial migration assay. Prior to experimentation, barrier integrity was confirmed using transendothelial electrical resistance (TEER), where monolayer resistance > 12Ω × cm^2^ dictated an intact monolayer. Endothelial monolayers were treated with inhibitors of endocytosis, followed by addition of live *T. pallidum.* Barrier permeability was assessed with a transwell solute flux assay, where endothelial permeability in the presence *T. pallidum* was monitored with a FITC-dextran fluorescent tracer by sampling from the bottom compartment of the transwell for fluorescence measurements throughout the time course of the experiments. *T. pallidum* traversal was evaluated by collecting the media from the lower compartment in each well and quantifying traversal with quantitative polymerase chain reaction (qPCR) detection of endoflagellar sheath gene *flaA*. Image created with BioRender. **(B)** *T. pallidum* traversal across HUVEC monolayers in untreated control (blue) or in the presence of inhibitors against lipid-raft mediated endocytosis (filipin, red), macropinocytosis (amiloride, yellow), dynamin-dependent endocytosis (Dynasore, green), or a positive control for endothelial barrier disruption that disrupts actin polymerization (cytochalasin D, purple). Endocytosis inhibitors were added at concentrations used in previous publications: filipin III at 5 μM (Rothberg et al., 1990; Schnitzer et al., 1994; Voigt et al., 2014), amiloride at 300 μM (Zhao et al., 2011; Li et al., 2013) (Sigma-Aldrich), Dynasore at 80 μM (Basagiannis et al., 2017; Loh et al., 2017), or Cytochalasin D at 0.1 μM (Wojciak-Stothard et al., 1999). Traversal was assessed using quantitative real-time PCR to measure *flaA* DNA concentrations in the bottom compartment of transwell plates. Results are presented as mean ± SEM percent *T. pallidum* traversal relative to the untreated control (set as 100% traversal, dashed red line) and statistical significance was analyzed by one-sample *t*-test for endocytosis inhibitor treatments; filipin (**p = 0.0157 *n* = 7); amiloride (ns *p* = 0.7017 *n* = 5); Dynasore (ns *p* = 0.4022 *n* = 6); cytochalasin D (***p = 0.00936 *n* = 4). **(C)** Solute permeability of HUVEC monolayers exposed to endocytosis inhibitors was evaluated with the fluorescent tracer FITC-dextran in the presence of *T. pallidum* and inhibitor treatments over 4 h. Results are normalized to transwells containing artificial basement membrane without endothelial cells (set as 100% flux of FITC-dextran) and presented as mean ± SEM. Statistical significance was analyzed by two-way ANOVA comparing no inhibitor control (*n* = 7), filipin (*n* = 7), amiloride (*n* = 5), Dynasore (*n* = 6), and cytochalasin D (*n* = 4). Comparison between inhibitor treatments ns *p* = 0.3073; comparison between time points *p* < 0.0001.

To investigate whether *T. pallidum* traverses endothelial barriers using a transcellular mechanism, HUVEC monolayers were treated with endocytosis inhibitors and treponemal traversal was monitored using qPCR on the lower chamber of transwell plates (Figure 8A). Endothelial cells were exposed to filipin, amiloride, or Dynasore which block lipid-raft/caveolae-mediated endocytosis, macropinocytosis, and dynamin-dependent endocytosis, respectively (Schnitzer et al., 1994; Koivusalo et al., 2010; Vercauteren et al., 2010; Preta et al., 2015). Cytochalasin D, which disrupts actin polymerization, was included as a positive control for barrier disruption. *T. pallidum* crossed HUVEC monolayers in all treatments; however, a statistically significant 60% reduction in *T. pallidum* transendothelial migration was observed in the presence of the lipid raft endocytosis inhibitor filipin (Figure 8B; ^∗∗^*p* = 0.0157), whereas no significant difference was observed in the presence of the macropinocytosis inhibitor (Figure 8B; amiloride; *p* = 0.7017) or the dynamin inhibitor (Figure 8B; Dynasore; *p* = 0.4022). As expected, the barrier disrupting agent cytochalasin D resulted in a significant increase in *T. pallidum* traversal (Figure 8B; cytochalasin D; ^∗∗∗^*p* = 0.00936*).* No significant difference in *T. pallidum* traversal was observed between untreated and Dynasore-treated HUVECs; however, the results were highly variable between experiments. This could be attributed to previously reported off-target effects of Dynasore, including disruption of actin dynamics (Gu et al., 2010) and lipid rafts (Preta et al., 2015). Control and inhibitor-treated HUVECs were also subject to a transwell solute flux assay to monitor changes in barrier permeability. No statistically significant difference in endothelial barrier permeability was observed between the no inhibitor control condition and the inhibitor treatments at any time point (Figure 8C *p* = 0.3073). As expected, a trend toward increased permeability was observed for the positive control cytochalasin D treatment condition; the incomplete barrier disruption observed is assumed to stem from the low cytochalasin D concentration used in the assay (0.1 μM compared to 5 μM used previously for barrier disruption) (Fong et al., 2015).

### Viability and Endothelial Attachment of *T. pallidum* and Adhesin Tp0751 Are Not Impacted by Endothelial Filipin Treatment

**FIGURE 9:**
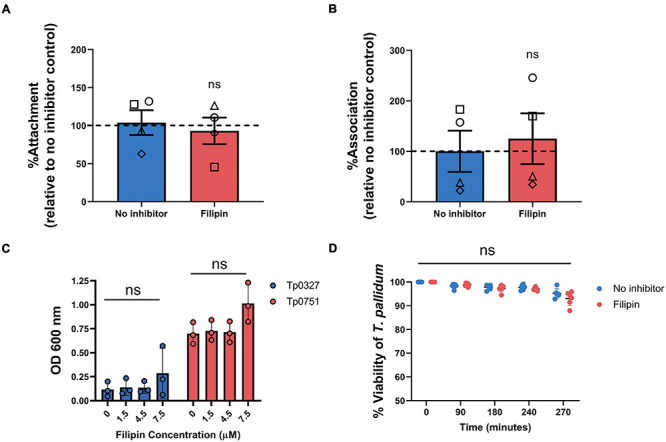
Filipin does not affect *T. pallidum* viability, endothelial attachment of *T. pallidum*, or endothelial attachment of the recombinant *T. pallidum* adhesin Tp0751. **(A)** Attachment of live *T. pallidum* to HUVEC monolayers treated with 5 μM filipin. Quantitative real-time PCR (qPCR) was used to measure *T. pallidum flaA* DNA concentration. Results were normalized to HUVEC *GAPDH* DNA concentration and presented as mean *flaA* copy number per picogram of *GAPDH* DNA from duplicate wells in four independent experiments. Data were normalized to the no inhibitor controls and presented as mean average %Attachment ± standard error of the mean. Individual data points represent independent experiments, and matched experiments are denoted by symbol type. Statistical significance was analyzed by Student’s *t*-test (*n* = 4, ns *p* = 0.6655). **(B)** Association of *T. pallidum* to HUVEC monolayers in transwell chambers treated with 5 μM filipin reported as percentage relative to the no inhibitor controls (normalized to 100%). From a subset of the traversal assays, HUVEC monolayers and associated *T. pallidum* were lysed, and qPCR was used to determine the *flaA*:*GAPDH* ratio of the DNA extracted from each sample. Monolayers were not subject to a washing step to remove non-adherent *T. pallidum* prior to sample lysis. Data were normalized to the no inhibitor control. Individual data points represent independent experiments, and matched experiments are denoted by symbol type; error bars represent SEM. Statistical significance was analyzed by Student’s *t*-test (*n* = 4, ns *p* = 0.7141) **(C)** Plate-based binding assay evaluating attachment of the recombinant *T. pallidum* adhesin, Tp0751, or negative control treponemal protein Tp0327 to HUVECs treated with different concentrations of filipin. Recombinant proteins were added in equimolar concentrations (15 μM), and results are presented as mean ± standard error of the mean from three independent experiments performed in triplicate. Statistical significance was assessed by one-way ANOVA (ns, *p* > 0.05). **(D)** Viability of *T. pallidum* cocultured with HUVECs after 30-min monolayer exposure to 5 μM filipin or no inhibitor control. Darkfield analysis of 10-μl samples was performed at 0, 90, 180, and 240 min from untreated or filipin-treated wells over a 4-h time course. At the experimental endpoint (270 min) each well was subject to trypsinization to dissociate *T. pallidum* and HUVECs. For each timepoint, treponemal motility was observed from five fields of view to a maximum of 200 treponemal cells. Results presented as three technical replicates from two independent experiments and statistical significance was assessed by two-way ANOVA (control vs. filipin; ns *p* = 0.2399).

We next sought to confirm that the lipid raft-mediated endocytosis inhibitor, filipin, was responsible for blocking *T. pallidum* endothelial traversal rather than endothelial adhesion. To explore the effects of this inhibitor on endothelial attachment, filipin treatments were incorporated into *T. pallidum* and Tp0751 adhesion assays. Pre-incubating HUVEC monolayers with filipin had no significant effect on live *T. pallidum* adhesion to monolayers (Figure 9A; *p* = 0.7341). To further confirm this finding, endothelial cell layers were isolated from no inhibitor control or filipin-treated monolayers from a subset of the *T. pallidum* traversal assays (Figure 8). Quantification of *T. pallidum* and HUVECs using qPCR demonstrated that there was no difference in *T. pallidum* association with endothelial cells between untreated or filipin-treated monolayers within the context of the transwell experimental setup (Figure 9B; *p* = 0.9262). Similarly, HUVEC pre-incubation with filipin did not affect endothelial attachment of the recombinant *T. pallidum* adhesin, Tp0751, or the negative control treponemal protein Tp0327 (Figure 9C; one-way ANOVA Tp0327, *p* = 0.342; Tp0751, *p* = 0.069). As previously reported (Lithgow et al., 2020), Tp0751 showed enhanced binding relative to the negative control protein, Tp0327, in all inhibitor conditions. Interestingly, HUVECs incubated with 7.5 μM filipin showed a slight upward trend in overall binding of both Tp0327 and Tp0751, but this increase was not found to be of statistical significance by one-way ANOVA. We further confirmed that none of the inhibitors affect HUVEC viability or induce cytotoxicity at the concentrations used in our experiments (Supplementary Figure 3). Finally, *T. pallidum* motility was monitored over a 4.5-h time course after coculture with untreated HUVECs or HUVECs exposed to filipin (5 μM) to discern whether filipin treatment of HUVEC monolayers could impact *T. pallidum* viability. No difference in *T. pallidum* motility was observed in samples taken from untreated or filipin-treated wells at any timepoint (Figure 9D; ns *p* = 0.2399), and normal cellular morphology was observed during motility assessment. These findings confirm that the viability of *T. pallidum* was not affected by filipin treatment (Figure 9D; ns *p* = 0.2399).

## Discussion

Vascular dissemination of *T. pallidum* is central to the disease progression of this highly invasive organism. The diverse clinical manifestations observed during secondary and tertiary syphilis can be attributed to the ability of *T. pallidum* to seed into numerous host tissues and organs (Mahoney and Bryant, 1934; Raiziss and Severac, 1937; Stokes et al., 1944; Cumberland and Turner, 1949). However, the specific molecular events that facilitate *T. pallidum* movement from the bloodstream into secondary infection sites are poorly understood, largely owing to the inherent difficulty of investigating this spirochete. The findings presented herein establish that Tp0751 and *T. pallidum* modify endothelial adherens junctions and that *T. pallidum* localizes to endothelial VE-cadherin at cell–cell borders. Further, we show that *T. pallidum* transendothelial migration occurs without disrupting barrier integrity and can be partially inhibited with an endocytosis inhibitor that targets raft-mediated endocytosis. These findings suggest that *T. pallidum* shares key elements of transendothelial migration with leukocytes and invasive extracellular bacteria.

**FIGURE 10:**
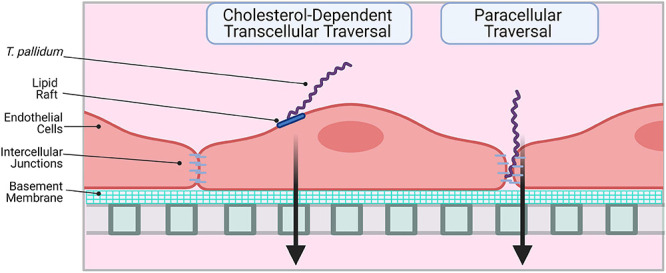
Proposed model: two routes for *T. pallidum* transendothelial migration. **Left:** *T. pallidum* coordinates transcellular traversal by engaging host receptors in lipid rafts (LamR) and inducing uptake and transport across the endothelium in a cholesterol-dependent manner. **Right:** *T. pallidum* adheres to endothelial cells at sites of intercellular junctions and disrupts VE-cadherin to open a paracellular traversal route. Intercellular junctions are re-formed after traversal to prevent vascular leakage. Figure created in BioRender.

This investigation reveals that *T. pallidum* localizes to intercellular junctions and modifies VE-cadherin junctional architecture; even with this junctional modification, *T. pallidum* traverses endothelial monolayers without disrupting barrier permeability. These findings corroborate previous observations that *T. pallidum* localizes to endothelial intercellular junctions and that *T. pallidum* transendothelial migration occurs without any discernable change in barrier permeability (Thomas et al., 1988, 1989). Paracellular transit may occur in a stepwise fashion, where adherens junctions are disrupted *via* progressive opening of junctions, followed by successive sealing of junctions after *T. pallidum* passage, similar to the mechanism used by leukocytes for paracellular traversal (Wettschureck et al., 2019). Taken together, these findings suggest that *T. pallidum* transendothelial migration is likely to occur *via* a mechanism that mimics leukocytes by transcellular traversal and/or paracellular traversal that is tightly regulated to prevent vascular leakage (Figure 10). In endothelial cells, similar host molecules are involved in, and similar mechanisms regulate, paracellular and transcellular traversal (Muller, 2013); thus, it is not surprising that leukocytes and invasive pathogens can utilize both traversal mechanisms. Although *T. pallidum* is considered to be an extracellular pathogen, electron micrographs have revealed instances of the spirochetes merging with endothelial cell surfaces (Wu et al., 2017) and residing within intracellular sites in epithelial cells and fibroblasts (Fitzgerald et al., 1975; Sykes and Kalan, 1975). While currently there is no definitive evidence to support an intracellular locale for *T. pallidum*, the possibility remains that a transient intracellular stage could exist during *T. pallidum* transendothelial migration.

Previous studies have revealed an *in vitro* traversal rate of 5.4% for *T. pallidum* transendothelial migration after 4 h (Thomas et al., 1988). Based on this finding, it was anticipated that the *in vitro* traversal rates of *T. pallidum* in our investigations would also be low. To ensure *T. pallidum* traversal was within the range of detection for darkfield microscopy and qPCR, we opted to add an inoculum of 3.2 × 10^7^ *T. pallidum* per well. Although this inoculum (9.7 × 10^5^ *T.* pallidum/mm^2^) is higher than what we have used for attachment and junctional modification assays (7 × 10^4^ *T. pallidum*/mm^2^), it is approximately 10-fold lower than what has previously been used for *T. pallidum* traversal assays (4 × 10^6^ *T. pallidum*/mm^2^) (Thomas et al., 1988). Quantitative analysis using real-time qPCR revealed that 1.35% of treponemes traversed HUVEC monolayers in untreated samples. The noted difference in traversal rate may be explained by experimental differences between the two studies; the previous study of the *T. pallidum* barrier traversal used a rabbit aortic endothelial cell line on gelatin-coated porous membranes with a significantly higher surface area, a higher starting inoculum of 5.0 × 10^8^ *T. pallidum*, and the less quantitative method of darkfield microscopy for treponemal quantitation (Thomas et al., 1988). However, one central commonality between these studies is that, despite the low traversal percentage noted when compared to the starting inoculum, a high number of *T. pallidum* are nevertheless traversing the endothelial barrier, with over 10^5^ *T. pallidum* traversing in the current study. Indeed, the low traversal rates are similar to what is seen with leukocytes, with the majority of leukocytes adhering to endothelial cells observed to re-enter the circulation (Muller, 2013).

The mechanisms of transendothelial migration characterized in other invasive bacteria have set a precedent for the disruption of intercellular junctions occurring in concert with transcellular traversal. *Neisseria meningitidis* traversal across the blood–brain barrier (BBB) occurs *via* a specialized transcellular route in which polarity complex and junctional proteins, including VE-cadherin, are recruited to sites of bacterial attachment, facilitating the opening of a transient traversal route below regions of meningococcal adhesion *via* the formation of new junctional sites. *In vitro*, this is accompanied by an increase in vascular endothelial barrier permeability resulting from depletion of junctional proteins from cell margins (Coureuil et al., 2009, 2010). Conversely, *in vivo* meningococcus invasion into the brain occurs without significant modulation of BBB permeability (Coureuil et al., 2017). Transcellular traversal of neuroinvasive *E. coli* K1 across the BBB occurs *via* caveolae-mediated uptake and is paired with disruption of adherens junctions and increased endothelial barrier permeability (Sukumaran et al., 2002; Sukumaran and Prasadarao, 2003; Mittal and Prasadarao, 2010; Krishnan et al., 2012; Salmeri et al., 2013). Similarly, *H. influenzae* traverses brain endothelial barriers *via* a transcellular route while disrupting VE-cadherin junctions and barrier permeability (Caporarello et al., 2018). Neuroinvasion of *S. pneumoniae* also occurs *via* transcytosis across brain endothelial cells, and while the specific mechanisms of traversal have yet to be delineated, numerous studies have reported that traversal occurs without disruption of intercellular junctions or barrier permeability (Ring et al., 1998; Iovino et al., 2013).

Previous studies suggest the existence of both paracellular and transcellular transendothelial migration for the invasive spirochete *Borrelia burgdorferi*. Live imaging of *B. burgdorferi in vitro* and *in vivo* demonstrate that the *B. burgdorferi* traversal mechanism mimics leukocyte paracellular transendothelial migration with a multistage adhesion process followed by localization to endothelial intercellular junctions and subsequent paracellular traversal (Moriarty et al., 2008, 2012; Norman et al., 2008). Further to this, *B. burgdorferi* does not appear to modify VE-cadherin junctional architecture (Sato and Coburn, 2017). Conversely, a transcellular traversal mechanism is supported by the observations of intracellular *B. burgdorferi* in endothelial cells (Comstock and Thomas, 1989; Ma et al., 1991; Wu et al., 2011). Similar to the findings presented for *T. pallidum* barrier traversal, *B. burgdorferi* localizes to intercellular junctions and transendothelial migration *in vitro* occurs without modulation of barrier permeability (Grab et al., 2005). It has been postulated that bacterial transendothelial migration could initially occur *via* transcellular routes, during which bacterial modulation of signaling pathways may induce opening of a paracellular route *via* junctional disruption (Dando et al., 2014). Further, the breakdown of paracellular to transcellular traversal usage *in vivo* is likely to be different than revealed by *in vitro* assays, reflecting the complex microenvironment faced by the pathogen *in vivo*. Indeed, studies measuring leukocyte barrier traversal showed that exposure of apical endothelial surfaces to chemokines increases the percentage of leukocytes undergoing transcellular traversal compared to paracellular traversal (Mamdouh et al., 2009).

Our findings demonstrate that *T. pallidum* transendothelial migration is significantly reduced by filipin III, which binds cholesterol in plasma membranes, disrupting lipid rafts and caveolae (Schnitzer et al., 1994). No significant difference in *T. pallidum* traversal was observed in the presence of amiloride which blocks macropinocytosis by inhibiting Na+/H+ exchange at the cell surface (Koivusalo et al., 2010). Similarly, no change in *T. pallidum* traversal occurred in the presence of Dynasore, which prevents both clathrin- and caveolar-mediated endocytosis by inhibiting the GTPase dynamin, required for vesicle fission in both of these endocytosis pathways (Preta et al., 2015). Endothelial caveolae are flask-shaped cholesterol-rich membrane microdomains, and caveolae-dependent uptake requires the GTPase dynamin to pinch off the newly formed endosome from the membrane (Li et al., 2005). Since a dynamin inhibitor did not reduce transendothelial migration, our results suggest that *T. pallidum* uses a cholesterol-dependent mechanism that does not rely on caveolae. Cholesterol-dependent invasion mechanisms have also been described for other bacterial pathogens: the processes of *Mycobacterium bovis* entry into macrophages (Gatfield and Pieters, 2000), *E. coli* invasion of mast cells (Shin et al., 2000), and *Campylobacter jejuni* epithelial invasion are all reduced with inhibitors targeting cholesterol (Wooldridge et al., 1996; Hu et al., 2006; Watson and Galan, 2008). Notably, while filipin III treatment reduced epithelial invasion of *C. jejuni* (Wooldridge et al., 1996; Hu et al., 2006), epithelial expression of a dominant-negative dynamin mutant did not impact *C. jejuni* invasion (Watson and Galan, 2008), demonstrating that *C. jejuni* also uses a cholesterol-dependent, dynamin-independent host invasion mechanism.

Collectively, these results suggest that, like other bacterial pathogens, *T. pallidum* may utilize lipid rafts as a portal for host cell invasion during the process of transcytosis. Lipid rafts are membrane microdomains that are enriched in cholesterol and sphingolipids that function as cell signaling platforms and can facilitate endocytosis. Intriguingly, we previously identified LamR as an endothelial receptor for *T. pallidum* (Lithgow et al., 2020), and numerous studies have demonstrated that LamR localizes to lipid rafts (Fujimura et al., 2005, 2006). However, *T. pallidum*-HUVEC adhesion/association was not affected by filipin treatment, demonstrating that filipin inhibits *T. pallidum* traversal rather than attachment. Thus, we propose that filipin blocks lipid raft-mediated recruitment of key signaling molecules required for *T. pallidum* traversal of endothelial cells.

There are several limitations to this study. First, the high concentration of the adhesin Tp0751 and the high numbers of *T. pallidum* used in the study may not be reflective of the *in vivo* situation and could led to an exaggerated disruption of endothelial junctions in our investigations. The true influence of *T. pallidum* on cell junctions may be more subtle, but regardless the overall phenomenon of *T. pallidum* paracellular transit would still be accomplished. Second, we did not investigate paracellular transport of *T. pallidum* directly. However, the collective observations of the co-localization of *T. pallidum* with VE-cadherin, the junction disruption induced by *T. pallidum*, and the incomplete abrogation of *T. pallidum* endothelial traversal upon pretreatment of endothelial cells with filipin suggest paracellular transport is contributing to *T. pallidum* endothelial traversal. Third, *T. pallidum* possesses cholesterol-rich membranes (Matthews et al., 1979), opening the possibility that filipin treatment of endothelial cells decreased treponemal traversal due a detrimental effect on *T. pallidum*. However, we did not observe any direct effect on *T. pallidum* morphology or motility (both indicators of viability) from pretreatment of HUVEC monolayers with filipin. Further to this, filipin treatment of HUVEC monolayers did not impair the endothelial adhesion of *T. pallidum* or the *T. pallidum* adhesin, Tp0751. These findings support the conclusion that filipin is blocking *T. pallidum* traversal, rather than affecting *T. pallidum* viability or adhesion.

In summary, the current study expands our understanding of *T. pallidum* traversal of endothelial barriers. We propose that *T. pallidum* uses a dual traversal strategy to cross endothelial barriers where transcellular traversal is facilitated by lipid rafts in a cholesterol-dependent manner and disruption of VE-cadherin at intercellular junctions supports paracellular traversal (Figure 10). This work reveals parallels between traversal mechanisms used by host leukocytes and other invasive pathogens. A detailed study of the molecular mechanisms facilitating *T. pallidum* lipid raft-mediated transcellular traversal, particularly in the context of traversal across the blood–brain and placental barriers, may reveal targets for infection intervention.

## Data Availability Statement

The raw data supporting the conclusions of this article will be made available by the authors, without undue reservation.

## Ethics Statement

The animal study was reviewed and approved by Animal Care Committee University of Victoria, Victoria, BC, Canada.

## Author Contributions

KL, LS, and CC contributed to the experimental design. KL, ET, ES, and AG conducted the experiments. KL, ET, ES, and CC were involved in the analysis and interpretation of the data. CC acquired financial support for the project. KL wrote the first draft of the manuscript with contributions from CC. ET, ES, AG, and LS reviewed the manuscript before submission for accuracy and intellectual content. All authors contributed to the article and approved the submitted version.

## Conflict of Interest

The authors declare that the research was conducted in the absence of any commercial or financial relationships that could be construed as a potential conflict of interest.
