# Supplementary material for: Treponema pallidum Disrupts VE-Cadherin Intercellular Junctions and Traverses Endothelial Barriers Using a Cholesterol-Dependent Mechanism
Source: Front Microbiol. 2021 Jul 20;12:691731. doi: 10.3389/fmicb.2021.691731 (PMC8329343; doi:10.3389/fmicb.2021.691731)
Supplement: Supplementary file 1 [file Data_Sheet_1.docx]

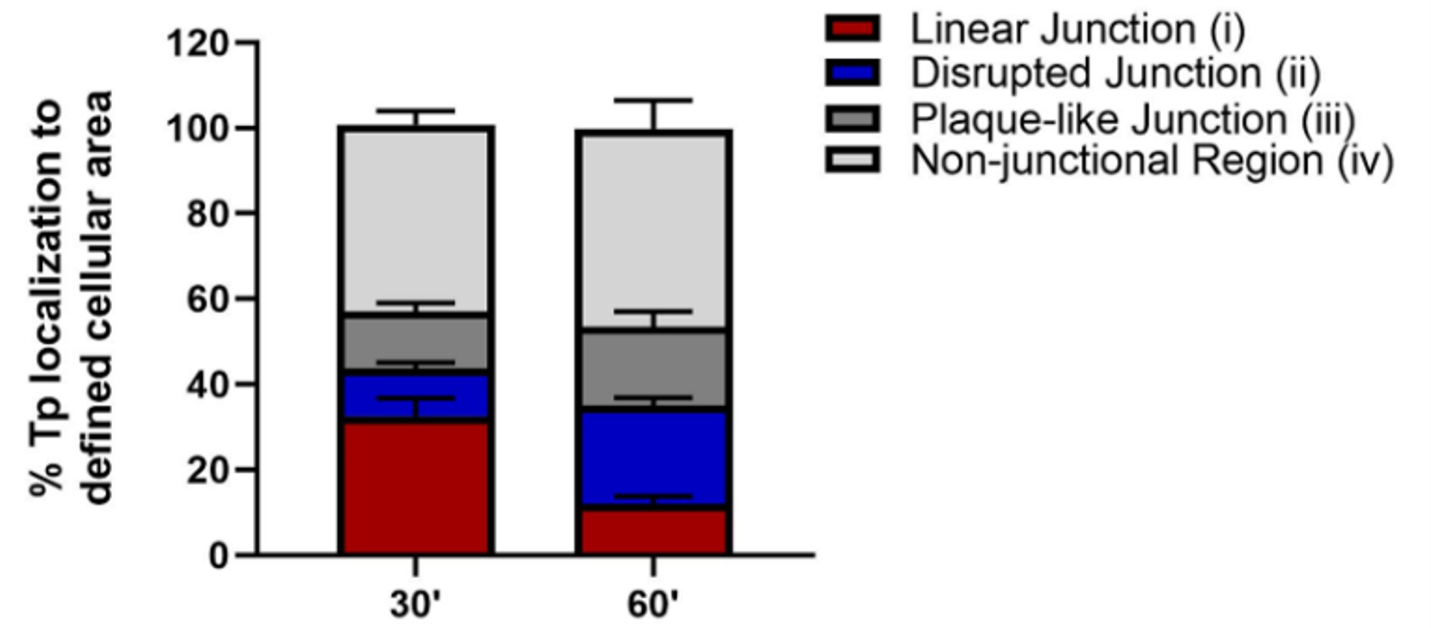


**Supplementary Figure 1. *Treponema pallidum* modifies endothelial VE-cadherin

architecture.** Percent localization of *T. pallidum* to defined cellular regions at 30 minute and 60

minute timepoints. Results presented as mean ± SEM from 3 independent experiments;

statistical significance was assessed with a two-way ANOVA and Sidak’s multiple comparison

test evaluating percent localization to cellular structures between 30 minute and 60 minute

timepoints for linear junction (* *p*=0.032), disrupted junction (* *p*=0.048), plaque-like junction (ns

*p*=0.91), and non-junctional area (ns *p*=0.99).


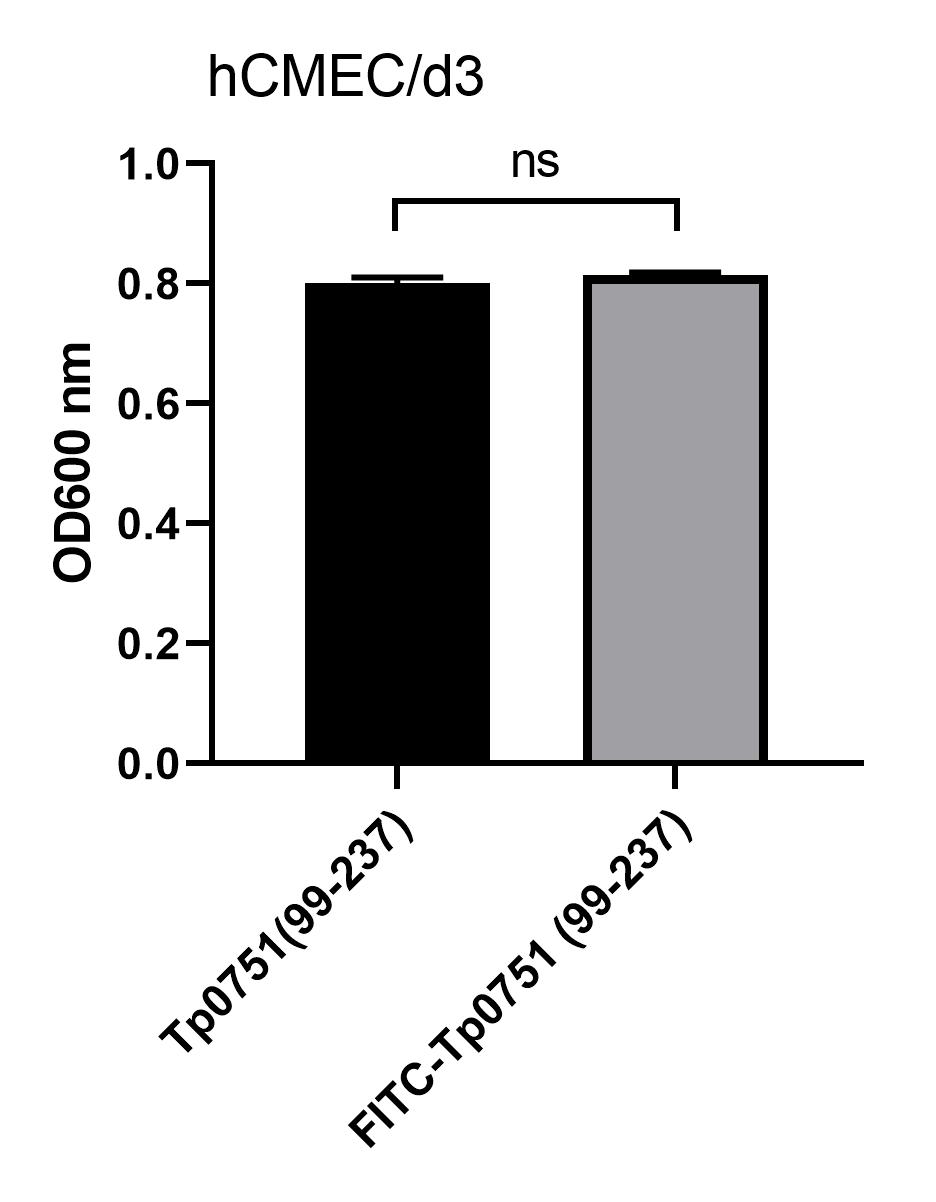


Supplementary Figure 2: Endothelial binding by Tp0751 is not affected by chemical labeling with FITC. Binding assays evaluating attachment of recombinant Tp0751 (V99-P237) or FITC-labelled Tp0751 (V99-P237) to a host cell monolayer of human cerebral microvascular endothelial cells (hCMEC/d3). Results are presented as mean absorbance at 600 nm ± SEM from triplicate wells in two independent experiments. Statistical analyses were performed using a Student’s *t-*test comparing endothelial binding of Tp0751 (V99-P237) to FITC-labelled Tp0751 (V99-P237) ns *p*=0.116.


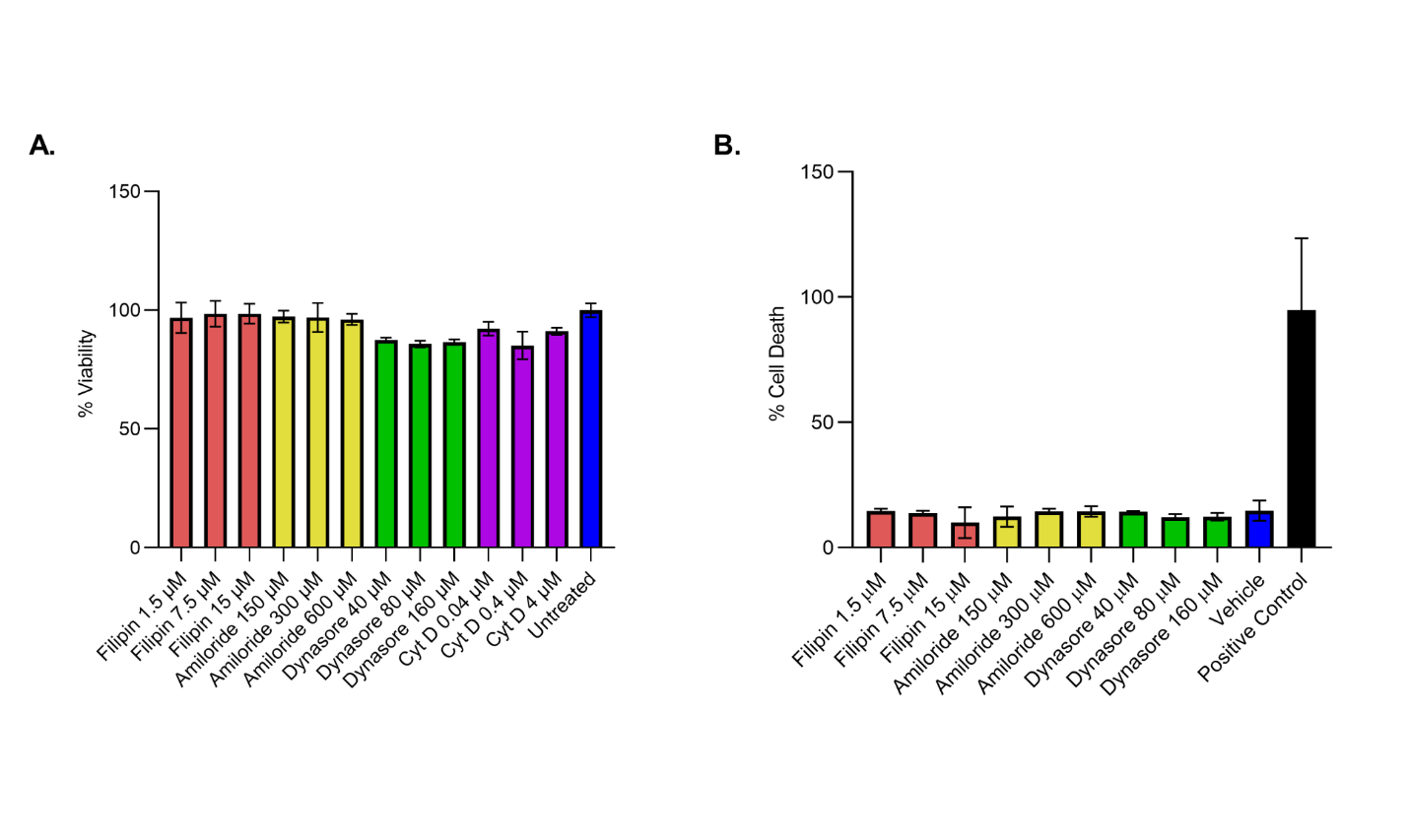
 **Supplementary Figure 3. Effect of endocytosis inhibitors on HUVEC viability.** **(A)** HUVEC viability was assessed by measuring ATP release using the CellTiter-Glo® luminescent assay. Endothelial cells were exposed to endocytosis inhibitors filipin, amiloride, Dynasore and cytochalasin D. Results are expressed as % viability relative to the untreated no inhibitor control (blue) from one independent experiment with three technical replicates **(B)** Cytotoxicity of endocytosis inhibitors was evaluated using the CellTox Green® assay. HUVECs were exposed to filipin, amiloride, or Dynasore at different concentrations. Results are normalized to the positive control (lysis buffer) and presented as percent cell death from one independent experiment with three technical replicates.
